# Endometriosis of the Bartholin gland: a case report and review of the literature

**DOI:** 10.1186/s13256-020-02424-7

**Published:** 2020-07-01

**Authors:** Tetske Heijink, Hein Bogers, Anneke Steensma

**Affiliations:** 1 Department of Gynaecology, Erasmus MC, Wytemaweg 80, Rotterdam, 3015 CN The Netherlands; 2 Department of Gynaecology, Sint Franciscus Gasthuis, Kleiweg 500, Rotterdam, 3045 PM The Netherlands

**Keywords:** Endometriosis, Bartholin’s gland, Vulva

## Abstract

**Background:**

In this case report we present a case of endometriosis in the Bartholin gland without surgery in the perineal area. So far, only five cases concerning endometriosis in the Bartholin gland, which may or may not be an isolated finding, have been reported in the literature.

**Case presentation:**

A 31-year-old Indo-surinamese woman with primary infertility presented at our out-patient clinic with cyclical vulvar pain. On gynecological examination, a cyst of 1 × 2 cm was found in the right gland of Bartholin. A transvaginal ultrasound revealed normal gynecological anatomy and did not reveal any contributing information. Due to the recurring pain of the cyst, surgery was scheduled.

During surgery, the marsupialization of the cyst resulted in drainage of a chocolate-colored fluid. Pathological examination revealed stroma lined with non-typical columnar epithelium with hemosiderin pigments, which confirmed a diagnosis of endometriosis in the Bartholin gland.

**Conclusion:**

Our findings revealed a case of endometriosis outside the pelvis, without any deep intraperitoneal involvement. So far, only five cases concerning endometriosis in the Bartholin gland, which may or may not be an isolated finding, have been reported in literature.

## Background

Endometriosis in the Bartholin gland without surgery in the perineal area is a rare presentation of extra-peritoneal endometriosis. So far, only five cases [1–5] concerning endometriosis in the Bartholin gland have been reported in the literature.

## Case presentation

A 31-year-old Indo-surinamese woman with primary infertility presented at our out-patient clinic with worsening cyclical vulvar pain during her menstruation for 3months. Her medical history included four spontaneous abortions and a diagnostic laparoscopy without any endometriosis lesions. Her family history did not mention any contributing diseases. On gynecological examination, a cyst of 1 × 2 cm was found in the gland of Bartholin on the right. A transvaginal ultrasound revealed normal gynecological anatomy and did not reveal any contributing information. Due to the recurring pain of the cyst, surgery was scheduled.

**Fig. 1:**
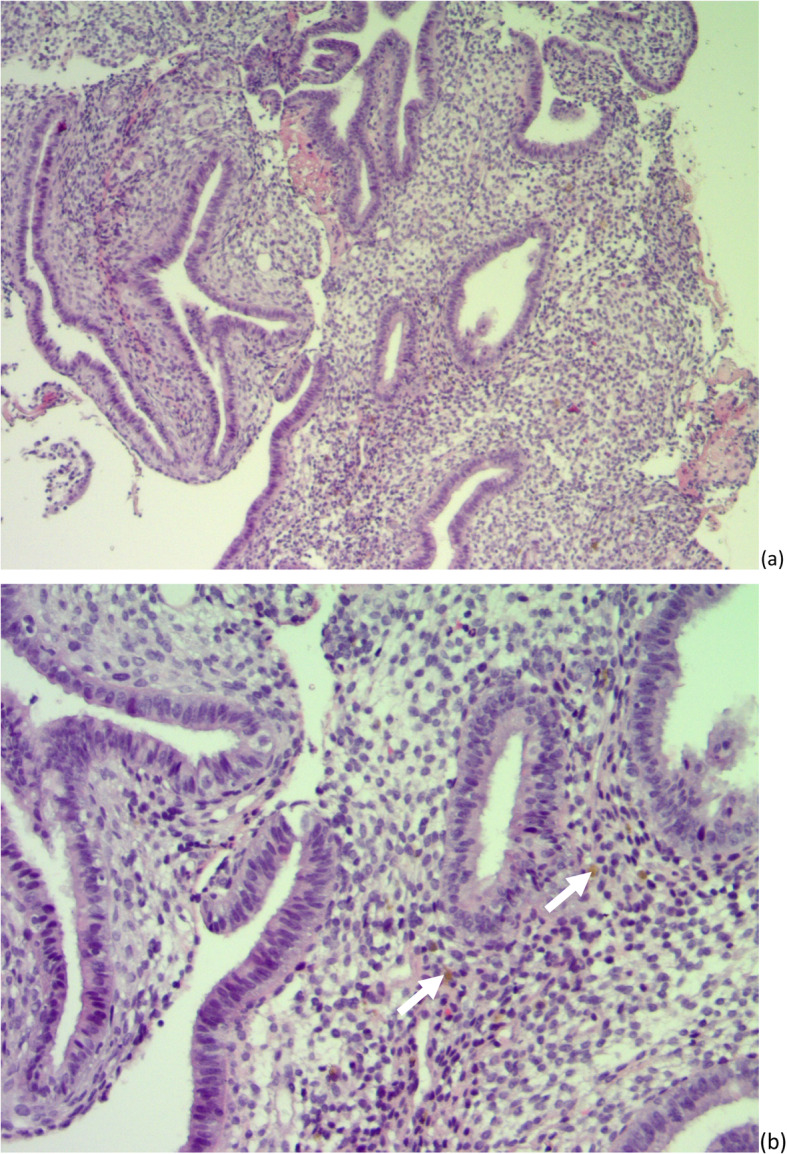
Endometrial glands and stroma. **a** Hematoxylin and eosin staining × 40. **b** Hemosiderin pigments (*arrow*), hematoxylin and eosin staining × 100

During surgery, the marsupialization of the cyst resulted in drainage of a chocolate-colored fluid. Pathological examination revealed stroma lined with non-typical columnar epithelium with hemosiderin pigments, which confirmed a diagnosis of endometriosis in the Bartholin gland (Fig. 1).

Further investigation to detect endometriosis in her pelvis included abdominal magnetic resonance imaging (MRI) according to our endometriosis protocol. The MRI did not reveal endometriosis that was left after the marsupialization of the gland of Bartholin. The MRI did show possible endometriosis in the sacro-uterine ligament, but showed no other signs of deep infiltrating endometriosis.

## Discussion

Bartholin glands are located symmetrically at the posterior section of the labia minora. The Bartholin gland plays a role in vaginal lubrication. Accumulation of mucus in the Bartholin gland, caused by an obstruction of a relatively small duct, leads to cyst formation of the gland. Enlarged Bartholin gland can have various diagnoses: non-inflammatory retention cyst, inflammatory retention cyst, chronic massive xanthomatous Bartholinitis, endometriosis, benign neoplasms, and malignant neoplasms [6]. A common complication of these cysts is an infection which is likely to result in Bartholin gland abscess, with *Escherichia coli* as a common bacterial pathogen for these abscesses [7].

Differential diagnosis of lesions which imitate diseases of the Bartholin glands should include different types of cysts, such as an inclusion, Gartner, Skene’s, sebaceous, vestibular mucosa, and canal of Nuck cyst. Other diagnoses to be considered are leiomyoma fibroma, hernia, hidradenoma, hematomas, lipomas, endometriosis, syringoma, accessory breast tissue, folliculitis, urethral diverticula, hidradenitis suppurativa, gonorrhea, syphilis, vaginitis, warts, or cancer [7]. Lesions should be suspected of being malignant when there are multiple recurrences of the lesions, lack of response to treatment, macroscopic solid lesions, and rapid growth [8, 9]. A biopsy is necessary to confirm a diagnosis of malignancy, because some malignant lesions are overlooked as a benign process [10].

Endometriosis is defined as endometrial glands and stroma outside the uterus. Endometriosis is a common disease, affecting 6–10% of women of childbearing age [11]. Endometriosis in the skin, pleura, lung, muscles, and abdominal wall have been reported. Perineal or vulvar lesions of endometriosis are usually associated with previous episiotomy. Endometriosis in the Bartholin gland seems not to be as rare as expected. Sosnik *et al.* examined 104 Bartholin glands. In two cases (2%), extrauterine endometriosis was diagnosed. Retention cysts were observed in 84.6%, in 47.7% of the cases inflammatory infiltration was observed and in nearly 3% benign and malignant neoplasms were reported [6].

However, so far only five cases concerning endometriosis in the Bartholin gland without perineal surgery have been reported in the literature (Table 1). In the reported cases the patients were between 28 and 34 years of age and, as shown in Table 1, none of them had had a vaginal birth. Most of the reported endometriosis lesions in Bartholin gland were treated by excision, one patient received adjuvant luteinizing hormone-releasing hormone (LHRH). None reported a recurrence of endometriosis in the Bartholin gland.

**Table 1:** Reported cases of endometriosis in the Bartholin gland

	Age	Location	Time of diagnosis	Treatment	Para	Other endometriosis spots
Robotti *et al.*, 2014 [5]	34	Left labium 3 cm	1 year after cesarean section	Unknown	1	Unknown
Hakimi *et al*., 2014 [3]	28	Left labium 6 × 4 cm	–	Excision, ruptured cyst, 6 month LHRH	0	Unknown
Aydin *et al*., 2011 [1]	28	Bilateral	2 weeks after cesarean section	Excision	1	Endometrioma ovarian cyst
Gocmen *et al*., 2004 [2]	31	Right labium majus; 3 × 5 cm	–	Excision	0	Lesions on the right uterosacral ligament
Matseoane *et al*., 1987 [4]	29	Left labium 3 × 4 cm	–	Excision	0	No

*LHRH* luteinizing hormone-releasing hormone

Endometriosis in the Bartholin gland may present itself with cyclic pain and swelling during menstruation. It presents as a dark red, brown, or blue cystic swelling at the posterior half of the vulva. Because endometriosis is associated with well-defined margins and corpuscular content, ultrasound can provide a diagnostic guidance for endometriosis in the Bartholin gland [5]. To confirm the diagnosis, a pathological examination is needed. Hemorrhage and hemosiderin deposits are important diagnostic features. As presented in Table 1, two of the cases did have other endometriosis lesions, so it is advised to conduct further diagnostic examination by MRI or laparoscopy.

The etiology and pathogenesis of endometriosis is still unclear, although thought to be complex and multifactorial. Several theories were hypothesized in the past. The three most common hypotheses are retrograde menstruation, metaplasia theory, and lymphatic or hematogenous spreading. Retrograde menstruation is an example of hematogenous spreading, it is reflux of endometrial cells through the fallopian tubes into the pelvic cavity during menstruation. In Meyer’s (1903) metaplasia theory, the development of the disease is a result of a transformation of peritoneal tissue into endometrial tissue through hormonal and immunological factors. Sampson’s (1925) theory explained the etiology of endometriosis by lymphatic and hematogenous spreading and transplantation of endometrial tissue. Modern theories of endometriosis pathogenesis are multifactorial; estrogens, genetics, direct transplantation, immune system, environment, and congenital defects may be involved [12].

After episiotomy, it is thought that mechanical transplantation of endometrial tissue into the scar causes perineal endometriosis [13]. Spontaneous spreading to the perineum is still unknown. Considering the localization of endometriosis in the Bartholin gland, direct transplantation in the gland during menstruation is a plausible explanation.

Treatment of perineal endometriosis is complete wide excision [14]. Treatment of endometriosis in the Bartholin gland is wide excision.

## Conclusion

A rare case of spontaneous endometriosis in the gland of Bartholin is presented in this case report. Even if there is no previous perineal surgery, perineal endometriosis in the gland of Bartholin should be considered when there is cyclic pain and swelling of the Bartholin gland. Endometriosis in the gland of Bartholin may or may not be an isolated finding.
